# Sustainable agronomic strategies and experimental design for Stevia cultivation in the high mountains region of Veracruz, Mexico

**DOI:** 10.1038/s41598-025-08267-w

**Published:** 2025-08-12

**Authors:** Carlos Iván Gutérrez-González, Eusebio Bolaños-Reynoso, José Luis Bolaños-Reynoso, Leticia López-Zamora, Juan Manuel Méndez-Contreras

**Affiliations:** División de Estudios de Posgrado e Investigación, Tecnológico Nacional de México, Instituto Tecnológico de Orizaba, Oriente 9, # 852, Emiliano Zapata Sur, 94320 Orizaba, Veracruz Mexico

**Keywords:** *Stevia*, *Steviol glycosides*, Stevia cultivation, Sustainable agriculture, Bioinsecticide, Agroecology, Biotechnology, Plant sciences, Climate sciences, Ecology, Environmental sciences, Analytical chemistry, Chemical engineering, Environmental chemistry, Process chemistry

## Abstract

*Stevia rebaudiana* Bertoni represents a sustainable alternative to conventional sweeteners due to its short growth cycle, low environmental impact, and potential for cultivation in diverse ecological regions. This study evaluated the growth performance of *Stevia* seedlings under controlled temperature and humidity conditions in the high mountain region of Veracruz, Mexico. A 2^2^ factorial experimental design was implemented to assess the effect of these variables on stem elongation, width, and leaf development. Results indicated that humidity had the most significant influence on plant growth, with optimal development observed under high humidity conditions (R²>0.85). Additionally, the application of *Azadirachta indica* (Neem) as a bioinsecticide demonstrated effective pest control without compromising seedling vigor, supporting its use as part of sustainable crop management. The findings highlight the potential of Stevia cultivation as a strategy for agroecological diversification and climate-resilient agriculture in Mexico. Future research should incorporate variables such as nutrient supplementation, light intensity, and microbial interactions to enhance predictive growth models and improve cultivation practices.

## Introduction

The increasing global demand for sustainable agricultural practices and natural alternatives to synthetic inputs has renewed interest in the cultivation of *Stevia rebaudiana* Bertoni^1^. As a non-caloric, plant-based sweetener, Stevia offers advantages over traditional sugar crops, including shorter growth cycles, lower water consumption, and reduced environmental impact^2^. In agricultural contexts, Stevia represents an opportunity for crop diversification, particularly in ecologically sensitive regions such as the high mountain areas of Veracruz, Mexico. Recent research demonstrated that the incorporation of *Stevia* in food products, such as dark chocolates, not only improves the nutritional profile but also enhances texture, thermal stability, and antioxidant properties, increasing its value as a functional food ingredient^3^.

Beyond its sweetening role, *Stevia rebaudiana* has also been recognized for the bioactivity of its industrial by-products. Compounds isolated from mother liquor sugar (MLS), a by-product of Stevia extraction, have shown potent nematicidal activity, suggesting additional agroecological applications in pest control^4^. Recent studies have explored the bioactive potential of Stevia extracts beyond sweetening, highlighting their role in sustainable agriculture. For instance, essential oils derived from Stevia rebaudiana have demonstrated insecticidal efficacy against Spodoptera frugiperda, offering an eco-friendly alternative for pest management in key crops^5^.

*Stevia rebaudiana* Bertoni, an herb from the Asteraceae family native to Paraguay, contains glycosides such as stevioside and rebaudioside A, responsible for its intense sweetness approximately 200–300 times greater than that of sucrose^6^. These compounds have been widely used as sugar substitutes in countries such as Japan, China, and South Korea^7^. In addition to its sweetening properties, *Stevia* has been associated with health-promoting effects, including antihyperglycemic, antihypertensive, anti-inflammatory, antitumor, and immunomodulatory activities^8,9^.

From a public health perspective, *Stevia* provides additional benefits compared to conventional sugars and synthetic sweeteners. It does not contribute to the formation of dental caries, a critical advantage over sucrose consumption, and is therefore recommended as a safer sweetening option for oral health^10^. Studies have also highlighted that compounds from *Stevia* may exert antimicrobial effects, further supporting its preventive role against dental diseases^10^.

Furthermore, recent evidence suggests that Stevia-derived compounds can influence metabolic processes beyond sweetening, with research indicating potential impacts on the biological activity of glycoproteins and metabolic regulation^11^. Notably, investigations on the non-nutritive sweetener rebaudioside A, a major component of *Stevia*, have revealed effects on microbial activity, including enhanced phage infectivity under certain conditions^12^.

Despite extensive research on its health benefits and industrial applications, relatively little attention has been given to *Stevia’s* agronomic performance under varying environmental conditions. Factors such as temperature, humidity, and light intensity are critical for optimizing early-stage seedling development, which influences overall yield and phytochemical composition. Innovative approaches, such as the application of LED lighting during in vitro culture, have demonstrated improvements in shoot multiplication and biosynthesis of steviol glycosides and phenolic compounds, contributing to sustainable intensification of Stevia production^13^.

Additionally, the integration of ecological pest management strategies, such as the use of Azadirachta indica (Neem) extracts as bioinsecticides, aligns with global efforts to reduce reliance on synthetic pesticides and promote agroecological farming systems^14,15^. Bioinsecticides based on natural products offer the advantage of being biodegradable, safe for non-target species, and environmentally friendly.

The high mountain region of Tequila, Veracruz, Mexico, was selected for this study due to its favorable agroclimatic conditions: an altitude of 1,623 m above sea level, an average annual temperature of 22 °C, and a relative humidity of 73%^16^. These conditions support the successful establishment and growth of Stevia seedlings, while promoting sustainable production systems.

Aligned with the United Nations’ 17 Sustainable Development Goals (SDGs), particularly those targeting health, sustainable agriculture, and responsible consumption, this study addresses critical global challenges: promoting health-focused nutrition, enhancing agricultural sustainability, and fostering environmental stewardship^1^. The plantation of *Stevia* in the high mountain regions of Veracruz has demonstrated strong adaptation, particularly when seedlings are cultivated in a substrate mixture of black soil and 30% gravel. This combination promotes optimal root aeration and drainage, enhancing root development and conserving soil moisture without requiring excessive irrigation. Regular irrigation of 700 mL per seedling every 2 days proved effective in maintaining hydration while minimizing water resource use.

Complementarily, sustainable pest control measures were implemented through the development of a laboratory-formulated bioinsecticide based on oak ash, vegetable oil, and potassium hydroxide. This natural formulation, together with the planting of Azadirachta indica specimens to continuously release azadirachtin, effectively controlled pest infestations in *Stevia* and *Coffea arabica* plants without compromising plant growth^15^. By bridging health, sustainability, and agronomic innovation, this research contributes meaningfully to the global transition toward agroecological practices that prioritize human well-being, biodiversity, and environmental resilience.

## Methodology

The methodology adopted in this study was designed to investigate the adaptation, growth, and protection of *Stevia rebaudiana* seedlings under controlled environmental conditions. The experimental design was structured into several key phases, as detailed below:

### Selection and preparation of seedlings

A total of 300 Stevia seedlings were selected from a reliable nursery in the state of Morelos, Mexico, ensuring uniformity in size (average heigh of 10–12 cm) and health. The seedlings were transplanted into an experimental field established at the facilities of the collaborating institution and at the experimental fields of the municipality of Tequila, Veracruz. To monitor environmental conditions, light intensity was measured using a digital lux meter (STEREN, model HER-020) equipped with a silicon photodiode sensor and optical filter, ensuring appropriate light exposure for optimal plant development. Additionally, protective infrastructure consisting of shade mesh and translucent polyethylene plastic was installed to regulate light exposure and minimize damage from excessive precipitation.

### Environmental conditions of the experimental site

The experimental study was conducted in the high mountain region of Tequila, Veracruz, Mexico, characterized by environmental parameters favorable for *Stevia* cultivation. The experimental fields are located at an altitude of 1623 m above sea level, with an average ambient temperature of 22 °C and a relative humidity of 73% during the study period, providing a stable environment for seedling development. Soil pH at the site was measured at 5.7, indicating slightly acidic conditions optimal for Stevia growth. Rainwater pH was determined to be 5.9. Light intensity at the cultivation site was recorded at 4,283 lx, measured using a previously calibrated digital luxometer (STEREN HER-020) equipped with a silicon photodiode sensor and optical filter, ensuring precise quantification of light exposure relevant to plant development. Additionally, dry bulb and wet bulb temperatures were recorded at 22 °C and 20 °C, respectively, reflecting a moderate temperature gradient and adequate atmospheric moisture, both crucial for promoting optimal physiological processes in Stevia seedlings. Overall, the environmental conditions of the experimental site provided a controlled and favorable setting for evaluating the effects of temperature and humidity on the growth parameters of *Stevia rebaudiana*.

### Experimental setup

Each seedling was transplanted into pots configured with a four-layered substrate: gravel (bottom layer), black soil, small stones, and a final mixture of soil and sawdust. This substrate design aimed to optimize root aeration drainage, and nutrient retention. Environmental humidity and temperature were continuously monitored using digital sensors to maintain optima conditions for growth. The substrate preparation was based on techniques analogous to those used in bioreactors and constructed wetlands for enhancing nutrient uptake and organic matter removal^14^.

### Bioinsecticide development and application

A natural bioinsecticide was formulated using oak ashes and corn oil, designed to offer effective pest control while promoting sustainable cultivation practices. Unlike conventional chemical insecticides, this formulation is biodegradable, less harmful to beneficial organisms, and cost-effective^14,15,18^. Applications were scheduled at critical stages of seedling development to prevent pest infestations while maintaining ecological balance.

### Monitoring of growth and environmental conditions

Seedling growth parameters including stem height, stem width, and leaf development were measured weekly using a caliper and ruler with millimetric precision. Climatic variables such as ambient temperature, humidity, and light intensity were recorded daily. Special attention was given to pest monitoring, with a focus on the control of Planococcus citri (cottony cushion scale), a prevalent pest known to affect tropical and subtropical crops^16,19^. Corrective actions, including bioinsecticide reapplication and irrigation adjustments, were implemented based on observed signs of stress or pest activity.

### Post-adaptation growth phase

Following the initial transplant shock period, seedlings were monitored to evaluate their adaptation and recovery. Growth responses were analyzed particularly during periods of significant climatic variability to assess resilience and optimize cultivation practices.

### Statistical analysis

Experimental data were analyzed using descriptive statistics (means and standard deviations) and factorial ANOVA to evaluate the effects of temperature and humidity on growth parameters. Statistical analyses were performed with significance set at *p* < 0.05. All statistical analyses were performed using Minitab 2023 software (Minitab, LLC, State College, PA, USA).

## Results

This section presents the outcomes of the factorial experimental design aimed at evaluating the influence of temperature and humidity on the growth parameters of *Stevia rebaudiana* seedlings. The analysis includes descriptive statistics, analysis of variance (ANOVA), regression modeling, and validation through normality plots. Key growth metrics such as stem length, stem width, and leaf development were assessed to understand the main effects and interactions of the environmental variables under controlled conditions. The findings provide insights into the environmental factors that most significantly impact Stevia cultivation, setting the foundation for optimized agronomic practices.

### Implementation of the experimental design

A 2^2^ factorial experimental design was implemented to evaluate the influence of temperature and humidity on the growth of *Stevia* seedlings. From an initial selection of 50 seedlings, 8 were randomly selected to form experimental groups. The experimental design variables and their corresponding measurements are summarized in Table 1.

**Table 1:** Variables and experimental desing values for *Stevia* cultivation Study.

X1	X2	Y1	Y2
Temperature	Humidity	Stem lenght	Stem width
(°C)	(%)	(cm)	(mm)
− 1	− 1	30	3.24
1	− 1	30.5	2.91
− 1	1	33	2.74
1	1	33	1.56
− 1	− 1	37	3.29
1	− 1	37	3.55
− 1	1	40	2.04
1	1	40	1.55

### Statistical analysis of stem length (Y1)

This section presents the statistical evaluation of stem length (Y1) as influenced by temperature and humidity. The analysis includes an analysis of variance (ANOVA), the construction of regression models, and a validation of model assumptions through normal probability plots. The findings aim to elucidate the primary environmental factors driving stem elongation under controlled cultivation conditions.

#### ANOVA analysis for Y1

The ANOVA results (Table 2) showed that both temperature and humidity had a statistically significant effect (*p* < 0.05) on stem length, explaining 95.11% of the total variability. Humidity exhibited a dominant effect.

**Table 2:** Variables and experimental desing values with respect to Y1.

Source	DF	Adj SS	Adj MS	F-value	*P*-value
Model	3	308.844	102.948	25.94	0.004
Linear	2	308.563	154.281	38.87	0.002
TEMP	1	38.281	38.281	9.65	0.036
HUM	1	270.281	270.281	68.10	0.001
2-term interactions	1	0.281	0.281	0.07	0.803
TEMP*HUM	1	0.281	0.281	0.07	0.803
Error	4	15.875	3.969		
Total	7	324.719			

**Fig. 1:**
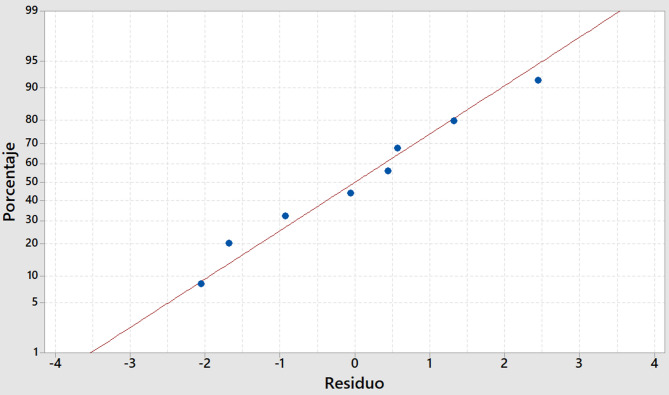
Normal probability Plot for Y1.

The Normal Probability Plot (Fig. 1) confirmed that the residuals were normally distributed.

#### Regression analysis for Y1

The factorial regression analysis yielded a high R² value of 99.32%. The regression equation (uncoded units) was:

$${\text{Y1 = 41}}{{.233 + 2}}.550{\text{(Temperature)}} + 7.550 {\text{(Humidity)}} - 0.718{\text{(Temperature }} \times {\text{Humidity)}}.$$

The positive coefficients for temperature and humidity reaffirm their constructive influence on stem length. However, the interaction term was not statistically significant (*p* > 0.05).

#### Refined model (excluding Interaction)

A refined model excluding the interaction term resulted in an R² of 98.52%, reinforcing that temperature and humidity were the principal factors influencing stem elongation:

$${\text{Y1 = 41}}.233 + 2.550({\text{Temperature}}) + 7.550({\text{Humidity)}}.$$

Table 3 shows the updated regression coefficients.

**Table 3:** Regression coefficients for Y1 (excluding interaction).

Term	Coefficient	SE coef	T-value	*P*-value
Constant	41.233	0.332	124.01	0.000
Temperature	2.550	0.332	7.68	0.002
Humidity	7.550	0.332	22.74	0.000

#### Practical implications for Y1

- Each 1 °C increase in temperature resulted in an average increase of 2.55 cm in stem length.
- Each 1% increase in humidity resulted in an average increase of 7.55 cm.
- Seedlings exposed to high temperature and high humidity (1,1) reached up to 51.47 cm compared to 29.67 cm under low conditions (− 1, − 1).

**Fig. 2:**
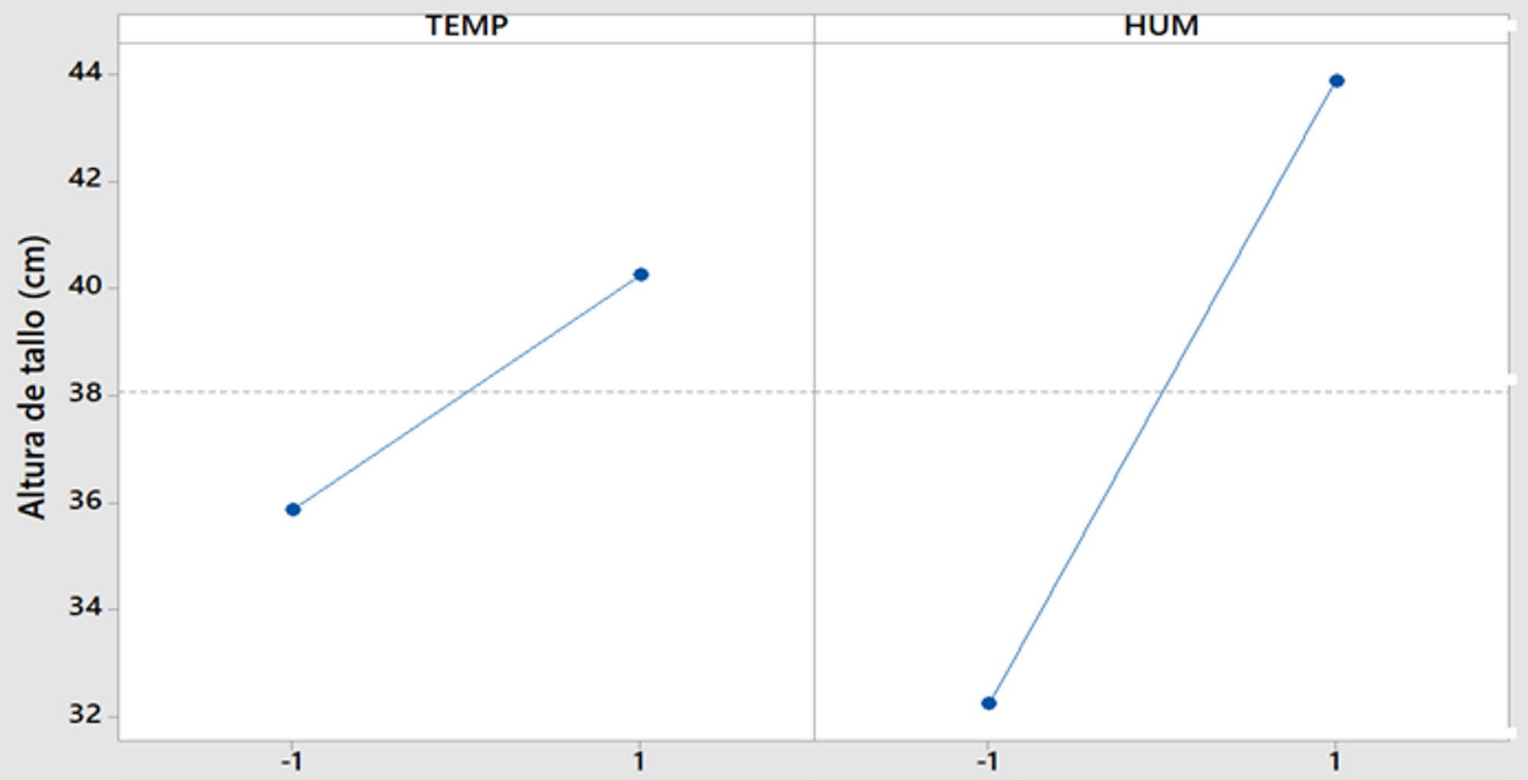
Factorial plot for temperature and humidity impact on stem length.

The factorial plot (Fig. 2) demonstrates that higher temperatures and humidity levels promoted greater stem elongation.

**Fig. 3:**
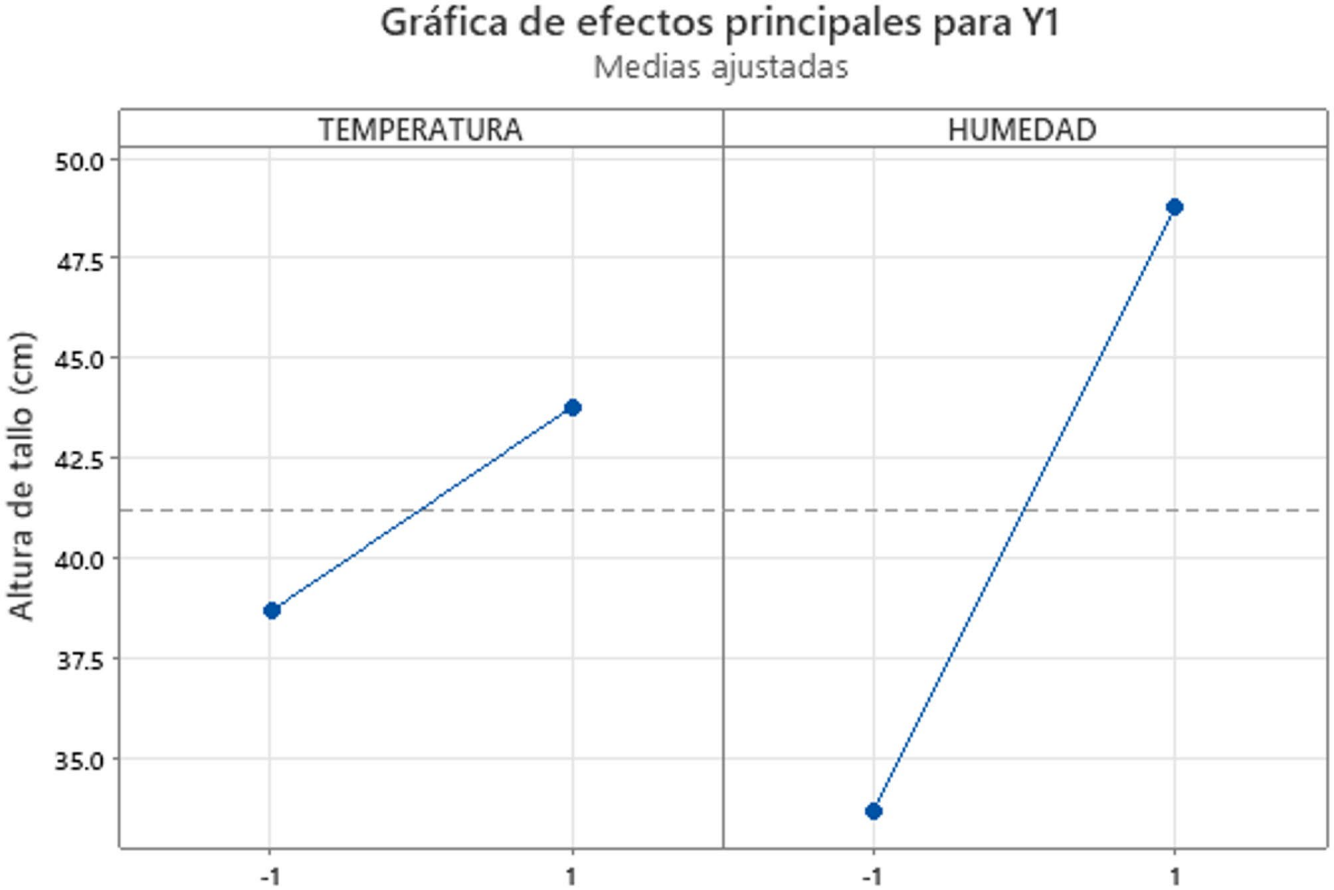
Main effects plot for Y1 (stem length).

The factorial plots (Figs. 2 and 3) confirm the predominant independent effects of temperature and humidity, with no significant interaction detected.

### Growth parameters: analysis of stem width (Y2)

This subsection focuses on the analysis of stem width (Y2) as a response variable to variations in temperature and humidity. ANOVA results and regression modeling are presented to determine the degree of influence of these environmental factors. Additionally, model adequacy and reliability are assessed to validate the predictive capacity for this morphological trait.

#### ANOVA and regression for Y2

The statistical analysis for Y2 (stem width) presented a moderate R² of 50.77% (Table 4), suggesting variability not fully explained by temperature and humidity alone.

**Table 4:** Variables and experimental desing values with respect to Y2.

Source	DF	Adj SS	Adj MS	F-value	*P*-value
Model	3	0.7177	0.2392	1.38	0.371
Linear	2	0.5347	0.2674	1.54	0.320
TEMP	1	0.3916	0.3916	2.25	0.208
HUM	1	0.1431	0.1431	0.82	0.416
2-term interactions	1	0.1830	0.1830	1.05	0.363
TEMP*HUM	1	0.1830	0.1830	1.05	0.363
Error	4	0.6960	0.1740		

Regression modeling revealed:

$${\text{Y}}2 = 4.901 + 0.439({\text{Temperature}}) + 0.786({\text{Humidity}}).$$

Tables 5 and 6 show the regression and ANOVA results respectively.

**Table 5:** Regression coefficients for Y2.

Term	Coefficient	SE coef	T-value	*P*-value
Constant	4.901	0.439	11.20	0.000
Temperature	0.878	0.439	4.01	0.014
Humidity	1.573	0.718	6.76	0.010

**Table 6:** ANOVA for Y2.

Source	DF	Adj SS	Adj MS	F-value	*P*-value
Model	2	6.48552	3.24276	28.70	0.002
Linear	2	6.48552	3.24276	28.70	0.002
Temperature	1	1.54001	1.54001	13.63	0.014
Humidity	1	4.94551	4.94551	40.81	0.010
Error	5	0.96496	0.19699		
Lack of fit	1	0.08611	0.08611	0.72	0.444
Pure error	4	0.87885	0.11971		
Total	7	7.05049			

The model validity was supported by the normality plot (AD = 0.136, *p* = 0.958).

### Analysis of leaf growth (Y3 and Y4)

The growth of leaves, both upwards (Y3) and downwards (Y4), was evaluated to gain a more comprehensive understanding of Stevia development under varying environmental conditions. Regression models and ANOVA analyses were performed separately for each parameter, with a focus on identifying significant factors and verifying model assumptions through normality tests.

#### Upward leaf growth (Y3)

The factorial regression showed an R² of 95.70%. The regression equation was:

$${\text{Y}}3 = 26.868 + 1.327({\text{Temperature}}) + 3.560({\text{Humidity}}).$$

The regression coefficients for upward leaf growth are summarized in Table 7, showing that humidity had a notably stronger effect than temperature.

**Table 7:** Regression coefficients for Y3.

Term	Coefficient	SE Coef	T-value	*P*-value
Constant	26.868	0.360	74.58	0.000
Temperature	1.327	0.360	3.68	0.014
Humidity	7.120	3.560	9.86	0.000

The ANOVA results for this model are presented in Table 8, confirming the statistical significance of the model and its predictors.

**Table 8:** ANOVA for Y3.

Source	DF	Adj SS	Adj MS	F-Value	*P*-Value
Model	2	115.487	57.743	55.61	0.000
Linear	2	115.487	57.743	55.61	0.000
Temperature	1	14.098	14.098	13.58	0.014
Humidity	1	101.389	101.389	97.64	0.000
Error	5	5.192	1.038		
Lack of fit	1	0.925	0.925	0.87	0.405
Pure error	4	4.267	1.067		
Total	7	120.679			

The normal probability plot confirmed model adequacy (AD = 0.254, *p* = 0.521).

#### Downward leaf growth (Y4)

The regression model for downward leaf growth yielded an R^2^ of 95.31%:

$${\text{Y}}4 = 22.015 + 1.238({\text{Temperature}}) + 2.887({\text{Humidity}}).$$

The regression coefficients for Y4 are presented in Table 9, highlighting the positive effect of both environmental variables.

**Table 9:** Regression coefficients for Y4.

Term	Coefficient	SE Coef	T-value	*P*-value
Constant	22.015	0.312	70.64	0.000
Temperature	1.238	0.312	3.96	0.011
Humidity	2.887	0.312	9.27	0.000

The corresponding ANOVA analysis is shown in Table 10, indicating a highly significant model fit.

**Table 10:** ANOVA for Y4.

Source	DF	Adj SS	Adj MS	F-value	*P*-value
Model	2	78.953	39.4763	50.81	0.000
Linear	2	78.953	39.4763	50.81	0.000
Temperature	1	12.251	12.2513	15.77	0.011
Humidity	1	66.701	66.7013	85.85	0.000
Error	5	3.885	0.7770		
Lack of fit	1	1.037	1.0376	1.46	0.294
Pure error	4	2.848	0.7120		
Total	7	82.837			

The normal probability plot validated the model (AD = 0.231, *p* = 0.709).

### Model refinement and final regression equations

After the initial regression modeling, further refinements were conducted to enhance predictive accuracy. Specifically, interaction terms that were found to be statistically non-significant (*p* > 0.05) were excluded from the models, resulting in simplified yet robust equations. The goal was to focus on the main effects of temperature and humidity, which consistently demonstrated significant impacts across all measured growth parameters.

For stem length (Y1), excluding the interaction term slightly reduced the R^2^ value from 99.32 to 98.52%, a minimal decrease that confirmed the primacy of the main factors. The final regression equation for stem length was:

$${\text{Y}}1 = 41.233 + 2.550({\text{Temperature}}) + 7.550({\text{Humidity}}).$$

Similarly, the regression equation for stem width (Y2) in uncoded units was determined as:

$${\text{Y}}2 = 4.901 + 0.439({\text{Temperature}}) + 0.786({\text{Humidity}}).$$

For upward leaf growth (Y3), the finalized regression model indicated:

$${\text{Y}}3 = 26.868 + 1.327({\text{Temperature}}) + 3.560({\text{Humidity}}).$$

And for downward leaf expansion (Y4), the final regression equation was:

$${\text{Y}}4 = 22.015 + 1.238({\text{Temperature}}) + 2.887({\text{Humidity}}).$$

In all cases, humidity exerted a greater positive effect than temperature, aligning with previous observations from the ANOVA results. The exclusion of interaction terms across models did not significantly impact predictive accuracy, indicating that temperature and humidity act largely independently on Stevia seedling development under the tested conditions.

### Broader implications of the results

The results of this factorial experimental design underscore the critical importance of environmental control in optimizing *Stevia* cultivation. Across all analyzed parameters (stem length, stem width, upward and downward leaf growth), humidity emerged as the most influential factor, consistently showing stronger coefficients and higher levels of statistical significance compared to temperature.

Stem elongation (Y1) and leaf expansion (Y3 and Y4) exhibited high coefficients of determination (R^2^ values above 95%), indicating that temperature and humidity were the principal environmental variables affecting *Stevia* growth under the tested conditions. In contrast, stem width (Y2) showed a slightly lower R^2^ value (91.99%), suggesting that this parameter may be more sensitive to additional or unmeasured factors, such as soil composition, nutrient dynamics, or intrinsic plant variability.

The factorial models developed demonstrated strong predictive capabilities, particularly for stem elongation and leaf expansion, providing valuable insights for agronomic management practices. These findings suggest that maintaining optimal humidity levels is crucial for promoting uniform and vigorous growth of *Stevia* seedlings, particularly during the early stages of development.

## Discussion

The factorial experimental design allowed a detailed analysis of how temperature and humidity influence the growth dynamics of *Stevia rebaudiana* seedlings. Across all analyzed parameters—stem length (Y1), stem width (Y2), and leaf growth both upwards (Y3) and downwards (Y4)—humidity emerged as the most significant factor, consistently presenting higher coefficients and greater statistical significance than temperature. The high R^2^ values obtained for Y1, Y3, and Y4 (> 95%) validate the robustness of the factorial models used, confirming that temperature and humidity are primary drivers of Stevia growth under controlled conditions. Stem elongation and leaf expansion demonstrated a clear positive response to increased humidity levels. However, stem width (Y2) exhibited slightly lower predictability (R^2^ = 91.99%), suggesting that additional factors, such as nutrient availability or plant genetics, may influence this growth aspect. The experimental data reaffirms the importance of maintaining optimal humidity conditions to promote vigorous and uniform development of Stevia seedlings. These results are highly relevant for agronomic management, especially in regions seeking to enhance the sustainable production of high-value crops while minimizing environmental impact. In addition to growth optimization, this study emphasized sustainable pest control through the use of natural bioinsecticides enriched with cactus mucilage and nettle extracts. These components are known for their natural repellent properties and were successfully integrated into pest management practices without adverse effects on seedling development. The effectiveness of such bioinsecticides represents a critical advance toward eco-friendly agriculture, aligning with international agendas such as the 2020–2023 Agricultural Sustainability Agenda and the UN SDGs. Moreover, the potential integration of advanced agricultural technologies—such as soil moisture sensors, climate-controlled chambers, and image-based growth monitoring systems—was identified as a key pathway for enhancing research precision and practical application in Stevia cultivation. Such tools could enable real-time data collection, improve irrigation efficiency, and further refine environmental control strategies. Overall, this study provides a valuable framework for promoting Stevia cultivation as a sustainable agricultural alternative, offering benefits in economic profitability, environmental conservation, and human health. The integration of sustainable practices demonstrated in this study offers a scalable model for the commercial expansion of Stevia cultivation under climate-resilient frameworks.

## Conclusions

This study highlights the critical role of environmental control in optimizing the growth of *Stevia rebaudiana* seedlings. The factorial experimental design demonstrated that humidity is the dominant environmental factor influencing all evaluated growth parameters,  showing consistently stronger positive effects compared to temperature. High humidity levels were particularly effective in promoting stem elongation and leaf expansion, which are key contributors to yield optimization. . The robust statistical models developed in this study, supported by high R² values for stem length, stem width, and leaf growth, confirm the predictive strength of the experimental design. These findings offer insights for the agroindustry, particularly for regions seeking to implement sustainable *Stevia* production systems aligned with global sustainability objectives. Futhermore , the use of natural bioinsecticides derived on cactus mucilage and nettle extract provide an effective enviromentally friendly pest control method, enhancing the ecological sustainability of Stevia cultivation.

## Data Availability

All data generated or analyzed during this study are included in this published article.
